# Temporal Analysis of Inflammatory Bowel Disease and Pancreatitis Co-Occurrence in Children and Adults in the United States

**DOI:** 10.14309/ctg.0000000000000628

**Published:** 2023-08-09

**Authors:** Ke-You Zhang, Ismaeel Siddiqi, Michelle Saad, Tatiana Balabanis, Melody S. Dehghan, Alexander Nasr, Vania Tolj, Aida Habtezion, K.T. Park, Maisam Abu-El-Haija, Zachary M. Sellers

**Affiliations:** 1 Department of Pediatrics, Division of Pediatric Gastroenterology, Hepatology, and Nutrition, Stanford University, Palo Alto, California, USA;; 2 Department of Medicine, Division of Digestive Diseases, University of Cincinnati, Cincinnati, Ohio, USA;; 3 Department of Pediatrics, Division of Pediatric Gastroenterology, Hepatology, and Nutrition, Cincinnati Children's Medical Center, Cincinnati, Ohio, USA;; 4 Department of Medicine, University of Cincinnati, Cincinnati, Ohio, USA;; 5 Department of Medicine, Division of Gastroenterology and Hepatology, Stanford University, Palo Alto, California, USA.

## Abstract

**INTRODUCTION:**

Pancreatitis in inflammatory bowel disease has been attributed to peripancreatic intestinal disease and/or drug-induced pancreatic toxicity. We used large cohort analyses to define inflammatory bowel disease and pancreatitis temporal co-occurrence with a detailed descriptive analysis to gain greater insight into the pathophysiological relationship between these 2 diseases.

**METHODS:**

Truven Health MarketScan private insurance claims from 141,017,841 patients (younger than 65 years) and 7,457,709 patients from 4 academic hospitals were analyzed. We calculated the prevalence of Crohn's disease or ulcerative colitis (UC) with acute pancreatitis or chronic pancreatitis (CP) and performed temporal and descriptive analyses.

**RESULTS:**

Of 516,724 patients with inflammatory bowel disease, 12,109 individuals (2.3%) had pancreatitis. Acute pancreatitis (AP) was 2–6x more prevalent than CP. In adults, AP occurred equally among Crohn's disease and UC (1.8%–2.2% vs 1.6%–2.1%, respectively), whereas in children, AP was more frequent in UC (2.3%–3.4% vs 1.5%–1.8%, respectively). The highest proportion of pancreatitis (21.7%–44.7%) was at/near the time of inflammatory bowel disease diagnosis. Of them, 22.1%–39.3% were on steroids during pancreatitis. Individuals with CP or recurrent pancreatitis hospitalizations had increased risk of a future inflammatory bowel disease diagnosis (odds ratio = 1.52 or 1.72, respectively).

**DISCUSSION:**

Pancreatitis in inflammatory bowel disease may not simply be a drug adverse event but may also involve local and/or systemic processes that negatively affect the pancreas. Our analysis of pancreatitis before, during, and after inflammatory bowel disease diagnosis suggests a bidirectional pathophysiologic relationship between inflammatory bowel disease and pancreatitis, with potentially more complexity than previously appreciated.

## Graphical abstract

**Figure:**
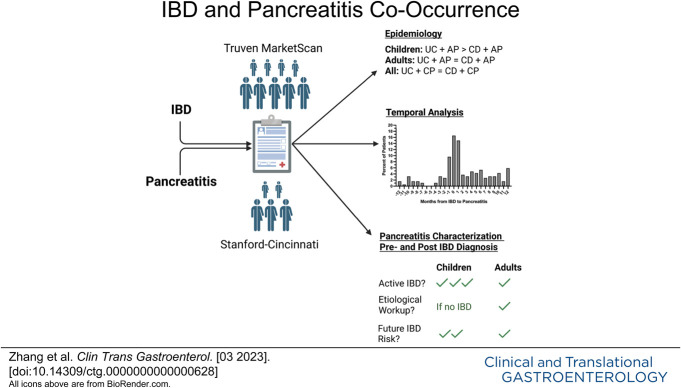

## INTRODUCTION

Inflammatory bowel disease (IBD) is a chronic inflammatory condition of the gastrointestinal tract that has been increasing in incidence, prevalence, and burden throughout the world (1). These global trends have been mirrored in the United States where Crohn's disease (CD) and ulcerative colitis (UC) are estimated to occur in 45.9 and 21.6/100,000 children and 197.7 and 181.1/100,000 adults, respectively, with a cost to the US health care system exceeding $25 billion per year (2). As many as half of all patients with IBD may exhibit at least 1 extraintestinal complication of IBD (3). Complications of the pancreas are considered to be relatively rare, with predominance in CD over UC (4,5). Despite its lower frequency compared with other complications of IBD, pancreatitis in IBD can create a conundrum for gastroenterologists in determining whether the pancreatitis is related to the pathogenesis of IBD, systemic inflammation, and/or an adverse effect of IBD medications, any of which may alter IBD treatment decisions and have significant short-term and long-term impact on patients.

Pancreatitis occurs with a prevalence of 12.3 and 111.2/100,000 in children and adults, respectively, for acute pancreatitis (AP) and 1.9 and 24.7/100,000 in children and adults, respectively, for chronic pancreatitis (CP) in the United States (6). While not common in absolute prevalence, pancreatitis can be debilitating with frequent and prolonged hospitalizations (7,8). Its potential impact on intestinal function, nutrition, intestinal microbiota, pain, and systemic inflammation can complicate the pathogenesis and/or treatment of other diseases in which it co-occurs, including IBD. Prior epidemiologic and descriptive studies in patients with co-occurrent IBD and pancreatitis have been limited to relatively small sample sizes and lack of uniformity of pancreatitis diagnosis, thereby limiting our understanding of the relationship between pancreatitis and IBD (9). The aims of this study were to (i) better understand the epidemiology of IBD and pancreatitis co-occurrence in children and adults, (ii) define the temporal relationship between IBD and pancreatitis, and (iii) characterize children and adults with co-occurrent IBD and pancreatitis diagnoses.

## METHODS

### Patient cohorts

**Figure 1.**
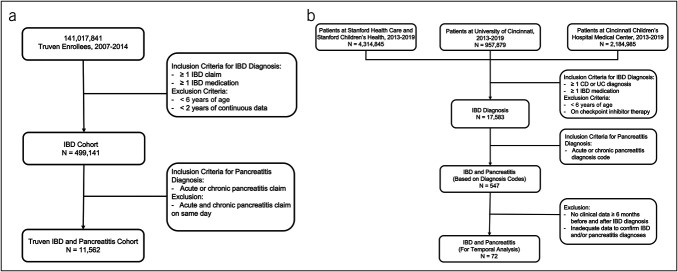
IBD and pancreatitis cohorts. (**a**) Truven MarketScan cohort. (**b**) Stanford-Cincinnati cohorts established by pooling individuals from Stanford Children's Health and Cincinnati Children's Medical Center (pediatrics), Stanford Health Care and University of Cincinnati (adults). The initial IBD and pancreatitis cohort was used for prevalence analysis, whereas the subsequent cohort was used for temporal analysis and detailed characterization. IBD, inflammatory bowel disease.

A retrospective cohort analysis was conducted under Institutional Board Approval. Deidentified insurance claims data were obtained from the Truven Health MarketScan Commercial Claims and Encounters database, which consists of outpatient, inpatient, and pharmaceutical claims of individuals with employer-sponsored insurance each year. Patients include active employees, early retirees, Consolidated Omnibus Budget Reconciliation Act recipients, and dependents aged 0–64 years from all 50 states of the United States. All enrollees with an inpatient or outpatient diagnosis of CD (555.X) or UC (556.X) between 2007 and 2014 were reviewed and assigned to the CD or UC group if they also had a claim for at least 1 CD or UC medication (10). These years were used based on institutional access to Truven Health MarketScan data. Indeterminate colitis (558.9) without CD or UC diagnoses were excluded. Those younger than 6 years were excluded from the analysis because very early–onset IBD generally represents a separate phenotype caused by underlying monogenic etiology or primary immune deficiency. Those with less than 2 years of continuous data surrounding their IBD diagnosis (<1 year prior and 1 year after) were omitted because the aim was to understand patient factors that were temporally associated with IBD and pancreatitis concurrent diagnoses. IBD diagnosis ± 1 year time frame was chosen to minimize subject dropout and plausibility of IBD and pancreatitis association (vs longer time frames). From the Truven IBD cohort, those with a diagnosis of AP (577.0) or CP (577.1) between 2007 and 2014 were selected, similar to our prior AP/CP epidemiological study using the same Truven dataset (Figure 1a). Patients who had both AP and CP on the same service date could not be classified concretely and were omitted from the analysis. This was previously shown to be 0.4% of pediatric pancreatitis and 0.4% of adult pancreatitis cohorts (6). However, patients with AP and then later developed CP were counted in each category. Time to first AP or CP diagnosis was used for analysis. To compare children and adults, pediatric groups were defined by ages 6–18 years and adult groups by ages older than 19 years.

The Stanford-Cincinnati cohort originated from the STAnford Research Repository database, which is composed of pediatric and adult data from Stanford Children's Health and Stanford Health Care, respectively, and the University of Cincinnati and Cincinnati Children's Medical Center electronic medical records system. Combining these 4 hospital datasets allowed us to compare pediatric and adult data from 2 different pediatric and 2 different adult hospitals in distinct regions of the United States. Data from 2013 to 2019 were selected based on feasibility to obtain complete electronic medical record access at each institution (based on dates of complete electronic medical record changes). Each research site deidentified data to assure patient confidentiality during analysis. For the Stanford-Cincinnati cohort, patients were included if they met the following criteria: (i) confirmed diagnosis of AP or CP (according to revised Atlanta and INternational Study group of Pediatric Pancreatitis: In search for a cuRE criteria) (11) and (ii) confirmed diagnosis of UC or CD (based on histopathology). For patients identified through diagnosis codes, similar international classification of diseases (*ICD*)–9 codes were used as with the Truven analysis. In addition, the following *ICD*-10 codes were used: CD (K50.X), UC (K51.X), AP (K85.X), and CP (K86.X). For temporal analysis, patients were required to have ≥6 months of continuous data before and after IBD diagnosis (Figure 1b). With the availability of more detailed data, a shorter period was used for this dataset. All eligible patients met this criteria. Given the smaller number of cases with IBD and pancreatitis in this cohort, we examined patients during the entire time window, not just 1 year before or after IBD. For epidemiological analysis, diagnostic categories were determined solely by diagnosis codes. For temporal analysis, all diagnoses were manually confirmed, and those not having sufficient documentation to confirm both IBD and pancreatitis diagnoses were excluded. Exclusion criteria included diagnoses of graft-versus-host disease, pancreatic cancer, or immune checkpoint inhibitor therapy.

### Data variables

The following variables were collected from the Stanford-Cincinnati cohort for analysis: demographic information, IBD subtype, date of first AP attack and (if applicable) subsequent attacks, date of IBD diagnosis (i.e. date of first endoscopy documenting IBD), endoscopy findings at IBD diagnosis, pancreatitis risk factors (alcohol use, gallstone-related disease, smoking), pancreatitis complications (fluid collections, necrosis, diabetes mellitus, exocrine pancreatic insufficiency (fecal elastase ≤100 μg elastase/g stool or on supplemental pancreatic enzymes), IBD complications (surgery, strictures, or fistulae), and extraintestinal IBD manifestations (arthralgia, uveitis, erythema nodosum, aphthous ulcers, pyoderma gangrenosum). Pancreatitis etiologies/risk factors were taken from medical records and not reassigned by the study investigators. Dates of use for corticosteroids, 5-aminosalicylic acids, thiopurines, and biologics within 90 days before an AP attack were examined. Pancreatitis severity was defined based on the revised Atlanta classification and North American Society for Pediatric Gastroenterology, Hepatology, and Nutrition Pancreas Committee guidelines (12,13). IBD severity was categorized into mild (none), moderate (1 or more criteria), and severe (2 or more criteria) based on the following: (i) anemia requiring blood transfusion, (ii) hospitalization for IBD flare, (iii) growth failure defined as loss of ≥10% of body weight or arrest of linear growth, or (iv) escalation of IBD therapy within 30 days of IBD diagnosis.

### Statistical analysis

Prevalence calculations for Stanford-Cincinnati cohort were based on all cases with IBD and/or pancreatitis regardless of the amount of data available. Temporality calculations were performed with time of IBD diagnosis as time zero. The first diagnosis of AP or CP was used to compare with time of IBD diagnosis. Differences between continuous variables were assessed using the Mann-Whitney *U* test, and differences between categorical variables were tested for significance with the χ^2^ test. Both univariable and multivariable conditional logistic regression models were performed to assess the odds ratios (ORs). Use of univariable or multivariable and continuous or categorical variables are indicated in the results section and/or figure legends. *P* values <0.05 were considered statistically significant for all analyses.

## RESULTS

### Description of cohorts

In the Truven cohort, from 141,017,841 patients, there were a total of 499,141 patients with CD or UC who met inclusion criteria, of whom 11,562 also had a diagnosis of AP and/or CP, resulting in an overall frequency of IBD and pancreatitis co-occurrence of 2.3% (Figure 1a). In the Stanford-Cincinnati cohort, from 7,457,709 patients, using a similar diagnosis code selection criterion, 17,583 met criteria for an IBD diagnosis between 2013 and 2019 (Figure 1b). Of them, 547 (3.1%) had a diagnosis of AP or CP. For temporal analysis, the Stanford-Cincinnati cohort was further refined to 72 individuals to ensure adequate pre-IBD and post-IBD diagnosis data were available. The characteristics of the Truven and Stanford-Cincinnati cohorts are summarized in Supplemental Table 1 (see Supplemental Digital Content, http://links.lww.com/CTG/A988). From these characteristics, significant differences between children and adults included the following: age, female predominance, increased body mass index in adults, a higher proportion of proximal gastrointestinal and rectal IBD pathology for patients with CD, and more acute recurrent pancreatitis in children vs more CP in adults. The Truven and Stanford-Cincinnati cohorts were largely similar, except for slightly younger age of children in the Stanford-Cincinnati cohort, greater percentage of AP in Stanford-Cincinnati adults, and less CP in both pediatric and adult Stanford-Cincinnati cohorts. Otherwise, all other characteristics were similar between the 2 cohorts.

We compared the prevalence of the co-existence of IBD and pancreatitis in both the Truven and Stanford-Cincinnati cohorts. The Stanford-Cincinnati diagnosis code cohort (identified by *ICD* 9/10 codes only) was used for a similar comparison with the Truven cohort. Across both datasets, AP occurred more commonly than CP (pediatric: 3.0–6.0 times, adult: 2.1–4.0 times). Within IBD subtypes, adult patients showed no difference in AP or CP prevalence between CD or UC. By contrast, pancreatitis occurred significantly more in children with UC than in those with CD, a phenomenon present in both the Truven and Stanford-Cincinnati datasets (Table 1).

**Table 1.** Prevalence of pancreatitis among patients with IBD based on ICD 9/10 diagnosis codes

	Truven cohort				Stanford-cincinnati diagnosis code cohort			
	Crohn's disease	Ulcerative colitis	*P* value (CD vs UC)	All patients^6^	Crohn's disease	Ulcerative colitis	*P* value (CD vs UC)	All Patients
Pediatric, n	19,990	10,935	—	107,290,008	1,799	756	—	2,589,478
AP, n (%)	290 (1.5)	248 (2.3)	**<0.001**	12,589 (0.01)	32 (1.8)	26 (3.4)	**0.013**	822 (0.03)
CP, n (%)	93 (0.5)	50 (0.5)	**1.0**	2,064 (0.002)	5 (0.3)	11 (1.5)	**<0.001**	223 (0.009)
*P* value (AP vs CP)	**<0.001**	**<0.001**	—	—	**<0.001**	**0.017**	—	—
Adult, n	204,983	263,233	—	285,238,034	6,291	8,737	—	4,919,096
AP, n (%)	3,669 (1.8)	4,145 (1.6)	**<0.001**	333,326 (0.12)	139 (2.2)	185 (2.1)	**0.676**	9,208 (0.19)
CP, n (%)	1,439 (0.7)	1,628 (0.6)	**<0.001**	78,625 (0.03)	59 (0.9)	90 (1.0)	**0.535**	4,684 (0.10)
*P* value (AP vs CP)	**<0.001**	**<0.001**	—	—	**<0.001**	**<0.001**	—	—
*P* value (pediatric apvs. adult AP)	**0.002**	**<0.001**	—	—	**0.298**	**0.020**	—	—
*P* value (pediatric CP vs. adult CP)	**0.001**	**0.183**	—	—	**0.010**	**0.194**	—	—

Bolded numbers indicate *P* values.

AP, acute pancreatitis; CD, Crohn’s disease; CP, chronic pancreatitis; n, number of individuals; UC, ulcerative colitis.

### Temporal relationship of IBD and pancreatitis Co-occurrence

**Figure 2.**
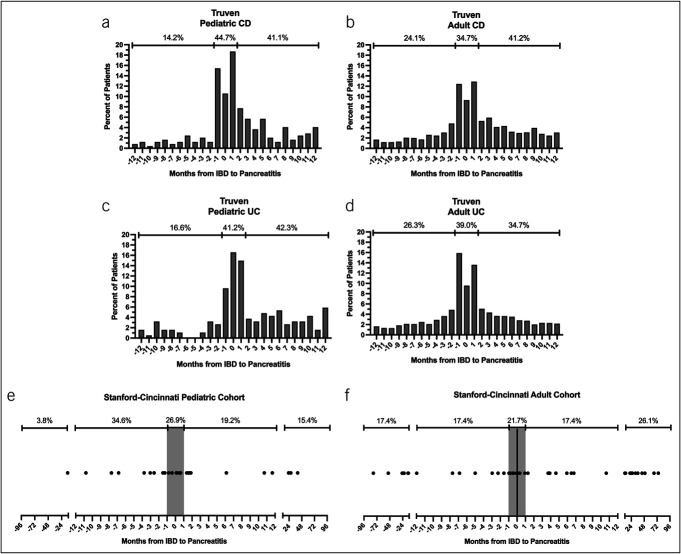
Temporal occurrence of pancreatitis in IBD. Time from IBD diagnosis to acute or chronic pancreatitis diagnosis was calculated and plotted for those who experience pancreatitis within 12–96 months of IBD diagnosis. (**a**)-(**d**) Analysis from Truven cohort. Columns represent percent of total patients at specific periods. Percentages indicate values from aggregate periods (−12 to −1, −1 to +1, +1 to +12 months from IBD to pancreatitis). (**e**) and (**f**) Similar to (**a**)-(**d**), except circles represent individual patients. Due to relatively small numbers, CD and UC data were combined. Gray column highlights −1 to +1 month period. CD, Crohn's disease; IBD, inflammatory bowel disease; UC, ulcerative colitis.

To better understand the relationship between pancreatitis and IBD, we examined the temporality of their co-occurrence. For this analysis, we set IBD as the anchoring point (time 0) and examined how many individuals developed any type of pancreatitis (AP or CP) within 1 year before and after IBD diagnosis. In all IBD cohorts, pancreatitis occurred most frequently at the same inpatient or outpatient encounter as their initial IBD diagnosis (time 0), with an incidence of 9.3%–16.2%. Similarly, a high proportion of pancreatitis diagnoses were found ±1 month of IBD diagnosis (Figure 2a–2d). In children, outside of this 1-month window, pancreatitis occurrence was steadily low before CD or UC diagnosis. In adult patients, pancreatitis before CD or UC diagnosis occurred more frequently than in children (*P* < 0.001) (Figure 2a–2d).

Within the Stanford-Cincinnati temporal analysis cohort, 85% of children with IBD and pancreatitis developed pancreatitis within 1 year of their IBD diagnosis, whereas 69% of adults developed pancreatitis within the same time frame. Pediatric and adult cohorts had mean (SD) time from IBD to pancreatitis of 2.9 (11.7) months and 4.2 (25.8) months, respectively (*P* = 0.818). Similar to the Truven cohort, both children and adults within the Stanford-Cincinnati cohorts showed a relatively increased percentage of pancreatitis ±1 month of IBD diagnosis (Figure 2e–2f). Detailed review of the Stanford-Cincinnati temporal analysis cohort revealed that 100 percent of children with IBD and pancreatitis had mild pancreatitis. Adults had significantly less mild disease, yet mild pancreatitis was still dominant (84.8%), with no episodes of severe pancreatitis. Consistent with this, there was a low rate of pancreatitis complications in both children and adults (Table 2). Exocrine pancreatic insufficiency rates were consistent with previous reports of exocrine pancreatic insufficiency rates in AP or IBD (14–16).

**Table 2.** Pancreatitis and IBD characteristics and complications in those with IBD and pancreatitis from the Stanford Cincinnati temporal analysis cohort

	Stanford-Cincinnati Temporal analysis cohort		
	Pediatric	Adult	*P* value
Pancreatitis severity			
Mild, n (%)	26 (100)	39 (84.8)	0.036
Moderate, n (%)	0 (0)	7 (15.2)	0.131
Severe, n (%)	0 (0)	0 (0)	1.0
IBD severity during first episode of pancreatitis			
Mild, n (%)	11 (57.9)	15 (45.5)	0.389
Moderate, n (%)	1 (5.3)	6 (18.2)	0.190
Severe, n (%)	7 (36.8)	12 (36.4)	0.977
Intestinal surgery, n (%)	6 (23.1)	18 (39.1)	0.167
Extraintestinal complications, n (%)	7 (26.9)	12 (26.1)	0.941
IBD medications within 90 d prior to AP			
Steroids	9 (34.6%)	16 (34.8%)	0.986
Aminosalicylates	15 (57.7%)	36 (78.3%)	0.065
Immunomodulators	6 (23.1%)	16 (34.8%)	0.301
Biologics	14 (53.8%)	28 (60.9%)	0.557
Pancreatitis complications (within 90 d of AP)			
Pancreatic fluid collection, n (%)	1 (3.8)	0 (0)	0.183
Pancreatic necrosis, n (%)	0 (0)	3 (6.5)	0.184
Diabetes mellitus, n (%)	0 (0)	2 (4.3)	0.284
Exocrine pancreatic insufficiency	3 (11.5)	10 (21.7)	0.279
IBD complications during pancreatitis			
Anal fissure, n (%)	1 (3.8)	1 (2.2)	0.692
Fistula, n (%)	1 (3.8)	3 (6.5)	0.630
Abscess, n (%)	5 (19.2)	12 (26.1)	0.508
Stricture, n (%)	6 (23.1)	7 (15.2)	0.403

AP, acute pancreatitis; IBD, inflammatory bowel disease; n, number.

*P* value column represents a comparison between pediatric and adult cohorts within each dataset.

### Pancreatitis within 1 month of IBD

**Figure 3.**
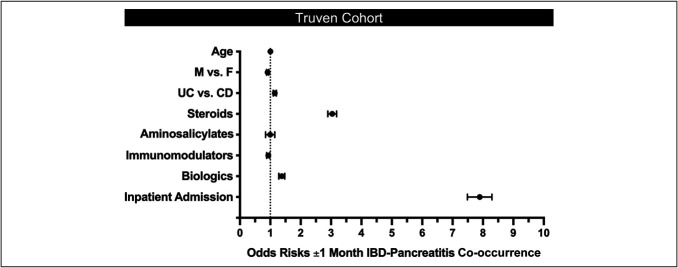
Risk factors of pancreatitis within 1 month of IBD diagnosis. Within the Truven cohort of IBD and pancreatitis individuals, the outcome of interest was a pancreatitis diagnosis within 1 month of IBD diagnosis (vs outside the 1-month window). Covariates used in multivariate analysis included age (continuous variable), sex, IBD diagnosis, IBD therapy classes (steroids, aminosalicylates, thiopurine immunomodulators, biologics), and IBD-associated hospital admission. Dotted line represents OR = 1 for comparison. IBD, inflammatory bowel disease.

With the peak incidence of pancreatitis typically occurring ±1 month of IBD diagnosis, we sought to better understand the characteristics of this cohort. For this analysis, we focused on the Truven cohort, given the larger dataset size (n = 2,371) (see Supplemental Table 2, Supplemental Digital Content, http://links.lww.com/CTG/A988). The mean age of both children and adults with pancreatitis within 1 month of CD or UC diagnosis was similar to that of the larger cohort of those with pancreatitis and IBD. There was a significantly higher percentage of female individuals in the adult cohort than in the pediatric cohort. Most of both children (88.5%) and adults (79.6%) within this subgroup of the Truven cohort were hospitalized for an IBD-related event during pancreatitis, and when we performed multivariate logistical regression to examine the factors associated with having IBD and pancreatitis within 1 month of each other vs further apart, steroids and inpatient admission were the highest associations (odds ratio [OR] = 3.03 and 7.89, respectively) (Figure 3). Biologic therapy, but not mesalamine or immunomodulator therapies, also slightly, but significantly, increased one's odds of being in this group.

### Pancreatitis after IBD

**Figure 4.**
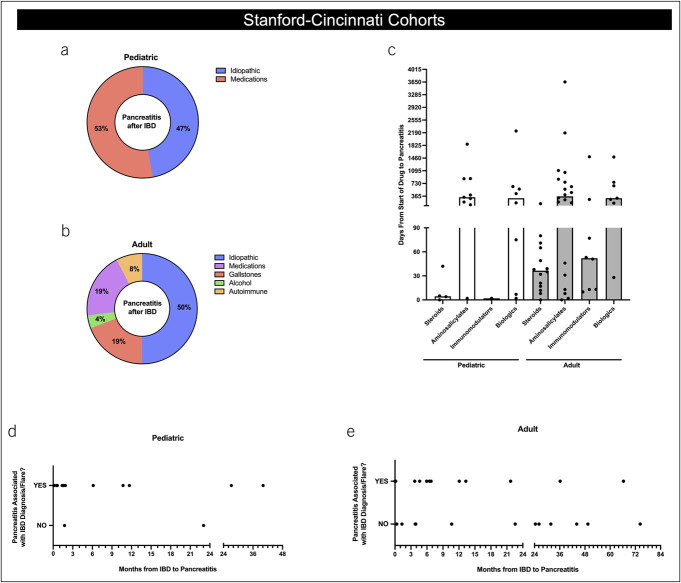
Characterization of individuals with pancreatitis after IBD diagnosis. (**a**) and (**b**) Breakdown (with percentages) of causes identified for pancreatitis in children **a** and adults **b** with IBD and pancreatitis in the Stanford-Cincinnati cohort. (**c**) Time delay from onset of IBD-related medications and diagnosis of pancreatitis in children and adults from the Stanford-Cincinnati cohort. Black circles represent distinct patients. Columns represent median values for each category. (**d**) and (**e**) Association of IBD activity in pediatric **d** and adult **e** patients with IBD from the Stanford-Cincinnati cohort that experienced pancreatitis after IBD. IBD, inflammatory bowel disease.

We next examined in more detail those individuals with pancreatitis after IBD to better understand the potential etiologies for pancreatitis in the presence of IBD. We primarily used the Stanford-Cincinnati cohort for this analysis because of our ability to extract detailed information not readily available and/or verifiable in the Truven dataset. We first examined documented risk factors for pancreatitis. In both children and adults, idiopathic and drug-induced pancreatitis predominated (Figure 4a and 4b). Not surprisingly, in adults, gallstone pancreatitis also accounted for a significant proportion of pancreatitis in those with IBD. In both children and adults, steroids and immunomodulators were taken for a median time of less than 60 days before pancreatitis, whereas aminosalicylates and biologics were approximately 300 days (Figure 4c).

Given the high rate of inpatient admissions for an IBD-related event in the pancreatitis within 1 month of IBD subgroup, we next examined whether IBD severity and/or IBD flares were associated with pancreatitis. In the Stanford-Cincinnati cohort, there was a bimodal distribution of IBD severity (either mild or severe) during pancreatitis in both children and adults (Table 2). Mild pancreatitis was evenly split between the pancreatitis before IBD and pancreatitis after IBD diagnosis subgroups (43.5% vs 56.5%, *P* = 0.378), but 89.5% of severe cases of pancreatitis occurred in the pancreatitis after IBD diagnosis group, compared with only 10.5% in the pancreatitis before IBD diagnosis group (*P* < 0.001). To further explore this association, we determined which of the pancreatitis episodes in the pancreatitis after IBD group occurred at the same time as an IBD flare (or initial IBD diagnosis). As shown in Figure 4d, in 87% (13/15) of children, the first pancreatitis episode in the pancreatitis after IBD subgroup was concurrent with “active” IBD, of whom, only 4/13 (31%) were on steroids during pancreatitis. By contrast, adults had similar proportion of individuals with pancreatitis regardless of “active” IBD, with 48% (11/23) active vs 52% (12/23) not active. This was significantly less than that in children (*P* = 0.016 vs children) (Figure 4e).

### Pancreatitis before IBD

**Figure 5.**
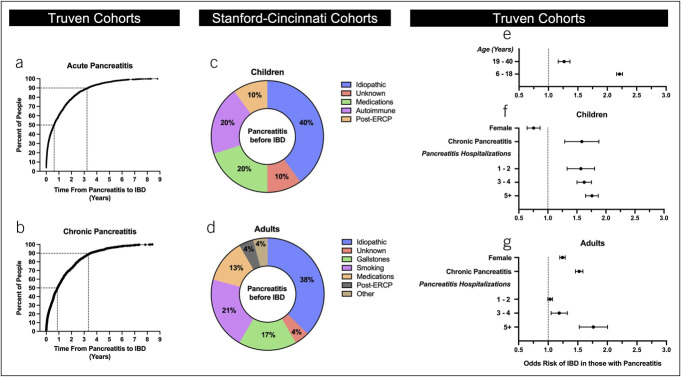
Characterization of individuals with pancreatitis before IBD diagnosis. (**a)** and (**b**) Time from AP **a** or CP **b** to IBD diagnosis in all individuals in the Truven cohort who were diagnosed with pancreatitis before IBD. Each dot indicates a distinct individual. Dotted lines indicate the amount of time that 50% or 90% of individuals were diagnosed with IBD after their pancreatitis diagnosis. (**c**) and (**d**) Breakdown (with percentages) of causes identified for pancreatitis in children **c** and adults **d** with a future IBD diagnosis in the Stanford-Cincinnati cohort. Causes were assigned based on original clinical documentation. “Idiopathic” category was used when a workup was performed, and this label was supplied by the clinical provider. “Unknown” category was used when no workup for causes was identified on review. (**e**)-(**g**) Within the Truven cohort of individuals with IBD and pancreatitis, the outcome of interest was an IBD diagnosis after pancreatitis diagnosis. Dots represent OR, and whiskers represent 95% confidence intervals. Dotted line indicates OR = 1 for comparison. (**e**) Univariable regression with age as the covariate (categorical variable). Individuals aged 6–18 and 19–40 years were compared with those aged 41–64 years. (**f**) and (**g**) Covariates used in multivariate analysis included sex (female vs male), pancreatitis diagnosis (AP vs CP), and total number of pancreatitis-related hospitalizations (compared with zero), which were compared among individuals in the Truven cohort with pancreatitis before IBD. AP, acute pancreatitis; CP, chronic pancreatitis; IBD, inflammatory bowel disease.

To gain insight as to whether there may be characteristics that predispose individuals with pancreatitis to develop IBD, we examined those individuals who were diagnosed with pancreatitis before an IBD diagnosis. Within the Truven cohort, 6.0% of children with a diagnosis of AP and 8.5% with CP later developed IBD. In adults, 3.0% with AP and 4.2% with CP went on to develop IBD. Pooling all individuals with AP or CP, the median time from AP or CP to IBD diagnosis was 227 or 328 days, respectively, with 90% of individuals being diagnosed with IBD approximately 3.5 years after pancreatitis diagnosis (Figure 5a and 5b). In contrast to the limited documented etiologies for pancreatitis in the pancreatitis after IBD group (Figure 4a and 4b), there was a more diverse list of etiologies in children and adults when pancreatitis was diagnosed before an IBD diagnosis (Figure 5c and 5d). We next performed univariable and multiple logistical regression models to determine what features of patients with pancreatitis might put them at risk of developing IBD. We first examined age by univariable analysis, comparing those 6–18 years of age with those 19–40 years and 41–64 years of age. The younger the cohort, the greater the risk; children with pancreatitis had an OR 2.21 of developing IBD vs OR 1.27 in those aged 19–40 years (Figure 5e). From multivariable analysis, CP inferred an increased risk that was similar for both children (OR 1.58) and adults (OR 1.52). Gender was also a risk factor; however, it differed between children and adults. In children, male individuals with pancreatitis were at higher risk of developing IBD with an OR 1.33 (95% CI: 1.14–1.54), whereas adult women were at increased risk with OR 1.24 (Figure 5f). Forty- to sixty-four–year-old women were at even higher risk than 19- to –36-year-old women, with an OR of 1.47 (95% CI: 1.37–1.59). Finally, we examined whether the number of hospitalizations for pancreatitis, as a marker for pancreatic disease activity, was a risk factor in developing IBD. We found that the more hospitalizations for pancreatitis one had, the greater the risk in developing IBD (OR 1.72, 95% CI: 1.51–1.96 for 5+ hospitalizations, all persons).

## DISCUSSION

Individually, IBD and pancreatitis cause significant morbidity, impaired quality of life for patients, and profound burden on the US health care system (2,17). When IBD and pancreatitis co-occur, these impacts may be magnified. The first suggestion of an association between pancreatic lesions and IBD was reported in 1950 by Ball et al who found 14% and 53% of gross and histologic pancreatic lesions, respectively, in an autopsy study of patients with UC (18). In the intervening 70 years, there have been a limited number of population-wide studies reporting the OR or annual incidence of AP in IBD. A recent meta-analysis summarizing 8 studies and 107,660 patients with IBD found a pooled OR of 3.11 for AP in IBD vs the non-IBD population (9). In our study, examination of 516,724 patients with IBD also identified an increased frequency of pancreatitis in IBD. In our current study, we identified that across all patients, pancreatitis occurred in approximately 2.3% of individuals with IBD. Previously reported rates for pancreatitis in IBD are typically approximately 1.5%–2% (0.06%–4.3%) (19–22). These low rates highlight the need for very large cohorts of patients to better understand the pathophysiologic basis of IBD and pancreatitis co-occurrence.

Extraintestinal manifestations of IBD are more prevalent post-IBD diagnosis compared with pre-IBD diagnosis (3). Post-IBD pancreatitis is typically considered to be gallstone related or drug related, with a minority of cases deemed idiopathic. In adults, where gallstone disease is highest, we found that post-IBD pancreatitis occurred more frequently than pre-IBD diagnosis. The rate of gallstone-induced pancreatitis in our adult multicenter cohort was 19%, slightly higher than has previously been reported in other IBD cohorts (7.5%–12.4%) (23–26). Even accounting for increased risk of gallstones and other comorbidities of IBD that may predispose to pancreatitis, AP remains increased in IBD (27). Nongallstone pancreatitis in IBD is commonly believed to be drug related; however, our data suggest the association between IBD and pancreatitis may not be so straightforward. The most striking finding from our study is that pancreatitis most frequently occurred at the time of, or in very close proximity to, IBD diagnosis. In addition, from our multicenter data, we found that post-IBD pancreatitis events were most commonly seen during an IBD flare or active disease, especially in children. This observation has been seen for other extraintestinal manifestations of IBD. In the Swiss IBD cohort study, the median length of time to first extraintestinal manifestation for pediatric patients was 1 month (28). Therefore, a major risk factor of pancreatitis in those with IBD may be the inflammatory state caused by active IBD. An alternative explanation could be that when individuals are presenting with suspected IBD, increased clinical workup (e.g. laboratory values and imaging) may increase the likelihood of detecting pancreatitis.

Corticosteroids are often a first-line treatment for severe IBD and are commonly associated with pancreatitis. In our study, despite a high rate of IBD-related hospitalization (80–90%) in those with pancreatitis ± 1 month of IBD diagnosis, only 22%–39% were on corticosteroids. We found similar rates in children who experienced pancreatitis during IBD flares (31%). In a study of 841 adults on corticosteroids (within 0–30 days), 19% had a history of AP (29). While we cannot exclude a potential role for steroid-induced pancreatitis, it may not account for most cases of IBD-associated pancreatitis in our cohort. Other medications commonly implicated in causing pancreatitis in patients with IBD, namely aminosalicylates and immunomodulators, did not incur increased risk of pancreatitis in those with pancreatitis ±1 month of IBD diagnosis. In our multicenter cohort, there was typically prolonged times from initiation of these drugs to pancreatitis (median length of time of approximately 30 days to >300 days). Thiopurine-induced pancreatitis occurs in 1.5%–7% of treated patients, usually within the first 30 days after the start of treatment (19). Biologic therapies had a small increased risk of pancreatitis; however, given the rarity in which biologic therapies have been described to cause pancreatitis, this finding may reflect the nature of IBD disease in these patients, which necessitates biologic therapy, rather than biologics themselves causing pancreatitis. We propose that medications may be more commonly assumed to be responsible for pancreatitis in IBD than may truly be responsible for pancreatitis. We observed that when pancreatitis occurred before an IBD diagnosis, there was a much more diverse set of causes attributed to the pancreatitis than if the pancreatitis was diagnosed after an IBD diagnosis. This was most profound for children. The combination of infrequent repeated exposure to potentially offending drugs, coupled with diseases that themselves may increase the risk of pancreatitis, makes it very difficult for gastroenterologists to tease out whether pancreatitis is caused by drugs, IBD, or both.

Pancreatitis in IBD has been described to be more prevalent in CD than UC (30), suggesting anatomical proximity and/or involvement of the ampulla as a nidus for pancreatitis. We did not see such a relationship in our study. In adults, there was no difference in pancreatitis rates whether one had CD or UC. We did observe more children with CD in the pancreatitis ±1 month of IBD diagnosis group in the Truven dataset; however, across all children, those with UC had higher rates of pancreatitis than those with CD, a finding also observed in the Stanford-Cincinnati cohort. In the latter, there was no correlation of duodenal/jejunal disease and pancreatitis. It remains unclear whether the mechanisms that drive pancreatitis in CD and UC are the same or divergent. There is intense interest in serum-based biomarker discovery to distinguish CD and UC (31–33). Furthermore, pediatric and adult disease are not equivalent. Intestinal disease in pediatric UC can be more extensive, severe, and aggressive than in adults, with 60%–80% of children presenting with pancolitis vs 20%–30% of adults (34,35). It is unclear whether circulating inflammatory cytokines or specific subsets of immune cells put the pancreas at risk of pancreatitis and whether these are the same for all individuals. It is also unclear whether there are differences in genetic risks (either in pancreas-specific or intestine-specific genes) that may predispose to developing pancreatitis in IBD. Further work in these areas is necessary but will likely require pooling large cohorts of patients with IBD, given the low prevalence of pancreatitis in IBD, heterogeneity between CD and UC, and potential differences between children and adults. While logistically challenging, this will likely lead to a substantial advancement in our understanding of the pathophysiology of pancreatitis in IBD.

We identified that IBD is not only a risk factor of pancreatitis but also that pancreatitis increased the risk of future IBD diagnosis in both children and adults. This was particularly true for younger individuals, male individuals, and those with multiple episodes of pancreatitis and a diagnosis of CP. In an Israeli cohort, Broide et al previously reported a higher percentage of AP preceding IBD in pediatric patients compared with that in adults, with a median time from AP to IBD of 6 months (22). In our cohort, most of the cases of IBD were diagnosed within 1 year of AP or CP. We do not know whether IBD was already present but not diagnosed during pancreatitis (36) or whether the pancreatitis incurred some other risk for developing IBD. It is also possible that imaging performed during pancreatitis diagnosis could increase the likelihood of finding IBD. There have been multiple studies examining how enteral nutrition may alter epithelial integrity and improve AP outcomes (37–40). Pancreatitis may also affect the intestinal microbiome (41), which very clearly affects IBD pathogenesis and course. As further research examines the potential link between pancreatitis and IBD, keeping IBD in the differential when evaluating abdominal pain and/or malabsorptive symptoms in those with a history of pancreatitis seems prudent.

### Limitations

Our current study provides a unique perspective on a long-standing problem of IBD and pancreatitis by combining large-scale commercial claims data with multicenter detailed cohort analysis. There are, however, several limitations that should be considered when interpreting our data. First, the Truven dataset, while nationwide, does not include those aged 65 years or older, those on Medicaid, or those on nonemployer-based insurance plans. Of note, approximately half of children and adults during the time frame of our study were on employer-based insurance plans (6). A notable limitation of our study is reliance on ICD 9/10 diagnosis codes for the largest set of patients for our analysis. The Truven MarketScan dataset has been used in numerous IBD studies. We attempted to mitigate misdiagnosis of IBD by combining an IBD diagnosis code plus use of an IBD medication, a strategy previously applied (10). We have previously documented the potential challenges of using the Truven dataset for pancreatitis (6); however, large datasets are required to study the co-occurrence of pancreatitis and IBD. For AP or CP, we did not have any additional laboratory work or imaging to confirm the diagnosis, although we have previously used the same dataset to examine AP and CP prevalence, with results similar to other studies (6). To compensate for this, we supplemented the Truven dataset with a multicenter cohort that would allow us to examine each patient's medical record for details not available in the Truven dataset. While these numbers are smaller and there were some differences between the cohorts, the Truven and Stanford-Cincinnati cohorts were generally in agreement, suggesting validity to the Truven data. Even with our multicohort analysis, we remain limited to extrapolating potential pathogenic factors rather than being able to establish cause and effect. However, it is our hope that with the increased insight from our data, some of which are consistent with smaller studies and some of which are different, we will be able to better design future studies aimed at biomarker discovery and/or new insights into disease pathogenesis for those with IBD and pancreatitis.

## CONFLICTS OF INTEREST

**Guarantor of the article:** Zachary M. Sellers, MD, PhD.

**Specific author contributions:** K.-Y.Z.: study design, data acquisition, data analysis, manuscript writing. I.S.: study design, data acquisition, manuscript editing. M.S.: data acquisition, manuscript editing. T.B.: data acquisition, data analysis, manuscript editing. M.S.D.: data acquisition, data analysis, manuscript editing. A.N.: data acquisition, manuscript editing. V.T.: data acquisition, manuscript editing. A.H.: study design and oversight, manuscript editing, funding. K.T.P.: study conceptualization, design and oversight, manuscript editing, funding. M.A.-E.-H.: study conceptualization, design and oversight, manuscript editing, funding. Z.M.S.: study conceptualization, design and oversight, manuscript writing and editing, funding.

**Financial support:** Personnel effort was supported by Stanford University (K.T.P., A.H., Z.M.S.) and the NIDDK (K08DK124684 to Z.M.S.; K23DK118190 to M.A.H.).

**Potential competing interests:** None to report.

**Study Highlights**

**WHAT IS KNOWN**

✓ Pancreatitis may occur in individuals with inflammatory bowel disease (IBD).
✓ The cause of pancreatitis in IBD is often assumed to be medication related.

**WHAT IS NEW HERE**

✓ Pancreatitis most often occurs at the time of IBD diagnosis and/or during IBD flares.
✓ Surrogates for IBD severity (steroids and/or biologic therapies and IBD-related hospitalizations) increased risk of pancreatitis.
✓ Reported etiologies for pancreatitis varied depending on whether pancreatitis was diagnosed before or after IBD.
✓ Chronic pancreatitis may increase future IBD diagnosis.

## Supplementary Material
