# Supplementary material for: Temporal Analysis of Inflammatory Bowel Disease and Pancreatitis Co-Occurrence in Children and Adults in the United States
Source: Clin Transl Gastroenterol. 2023 Aug 9;14(11):e00628. doi: 10.14309/ctg.0000000000000628 (PMC10684167; doi:10.14309/ctg.0000000000000628)
Supplement: Supplementary file 1 [file ct9-14-e00628-s001.docx]

**SUPPLEMENTAL TABLES**

**Supplemental Table 1. General Description of Children and Adults with both IBD and Pancreatitis**

|  | **Truven Cohort** | | | **Stanford-Cincinnati Temporal Analysis Cohort** | | |
| --- | --- | --- | --- | --- | --- | --- |
|  | **Pediatric** | **Adult** | **P Value** | **Pediatric** | **Adult** | **P Value** |
| **Unique Patients, n** | 681 | 10,881 | - | 26 | 46 | - |
| **Mean (SD) Age at Pancreatitis Diagnosis (Years)** | 14.6  (2.9) | 45.6 (11.9) | <0.001 | 12.7^a^  (3.6) | 44.6 (16.7) | <0.001 |
| **Sex, n (%)** |  |  |  |  |  |  |
| **Female** | 332 (48.8) | 6,322 (58.1) | <0.001 | 9  (34.6) | 25 (54.3) | 0.108 |
| **Male** | 349 (51.2) | 4,559 (41.9) | <0.001 | 17  (65.4) | 21 (45.7) | 0.108 |
| **BMI (kg/m^2^)** | - | - |  | 18.9  (4.4) | 27.4 (7.7) | <0.001 |
| **Ethnicity, n (%)** |  |  |  |  |  |  |
| **Hispanic** | - | - |  | 4  (15.4) | 3  (6.5) | 0.101 |
| **Non-Hispanic** | - | - |  | 22  (84.6) | 43 (93.5) | 0.101 |
| **Race, n (%)** |  |  |  |  |  |  |
| **Asian** | - | - |  | 0  (0) | 5  (10.9) | 0.081 |
| **Black** | - | - |  | 2  (7.7) | 6  (13.0) | 0.491 |
| **Hispanic/Latinx** | - | - |  | 4  (15.4) | 3  (6.5) | 0.221 |
| **White** | - | - |  | 16  (61.5) | 31  (67.4) | 0.614 |
| **Other** | - | - |  | 4  (15.4) | 1  (2.2) | 0.035 |
| **IBD Subtype, n (%)** |  |  |  |  |  |  |
| **Crohn’s Disease** | - | - |  | 14  (53.8) | 25 (54.3) | 0.967 |
| **Esophagus** | - | - |  | 9  (64.3) | 1  (4.0) | <0.001 |
| **Stomach** | - | - |  | 12  (85.7) | 1  (4.0) | <0.001 |
| **Duodenum** | - | - |  | 6  (42.9) | 2  (8.0) | 0.010 |
| **Jejunum** | - | - |  | 0  (0) | 7  (28.0) | 0.029 |
| **Ileum** | - | - |  | 8  (57.1) | 10  (40.0) | 0.304 |
| **Colon** | - | - |  | 11  (78.6) | 22  (88.0) | 0.435 |
| **Rectum** | - | - |  | 10  (71.4) | 2  (8.0) | <0.001 |
| **Ulcerative Colitis** | - | - |  | 12  (46.2) | 21  (45.7) | 0.967 |
| **Pancreatitis Subtype, n (%)** |  |  |  |  |  |  |
| **Acute Pancreatitis** | - | - |  | 19  (73.1) | 40^c^ (87.0) | 0.141 |
| **Chronic Pancreatitis** | - | - |  | 0 ^b^  (0) | 4^d^  (8.7) | 0.122 |
| **Acute Recurrent Pancreatitis** | - | - |  | 7  (26.9) | 2  (4.3) | 0.005 |
| **Geographic Region (%)** |  |  |  |  |  |  |
| **Northeast** | 186 (27.3) | 2,514 (23.1) | 0.012 | - | - |  |
| **North Central (Midwest)** | 157  (23.0) | 2,448 (22.5) | 0.762 | - | - |  |
| **South** | 214 (31.4) | 3,950 (36.3) | 0.001 | - | - |  |
| **West** | 100 (14.7) | 1,697 (15.6) | 0.523 | - | - |  |
| **Unknown** | 24  (3.5) | 272 (2.5) | 0.109 | - | - |  |

*IBD: Inflammatory Bowel Disease, SD: Standard Deviation, n: number. P value column represents a comparison between Pediatric and Adult cohorts within each dataset. a, P=0.001; b, P=0.012; c, P=0.008; d, P<0.001 vs. same category in Truven cohort.*

**Supplemental Table 2. Characteristics of Children and Adults from Truven Cohort with Pancreatitis within 1 Month of IBD Diagnosis**

|  | **Pediatric:**  **IBD Time 0 ± 1 Month**  **(n=187)** | **Adult:**  **IBD Time 0 ± 1 Month**  **(n=2,184)** | **P Value** |
| --- | --- | --- | --- |
| **Mean Age in Years (SD)** | 14.7 (2.7) | 44.7 (14.0) | <0.001 |
| **Female, n (%)** | 87 (47.0%) | 1,192 (54.6%) | 0.048 |
| **Patients with CD, n (%)** | 107 (43.0%) | 882 (33.1%) | <0.001 |
| **Patients with UC, n (%)** | 74 (37.4%) | 1,194 (37.1%) | 0.365 |
| **Patients with AP, n (%)** | 147 (41.5%) | 1,556 (37.3%) | <0.001 |
| **Patients with CP, n (%)** | 29 (37.2%) | 454 (28.8%) | 0.030 |
| **Patients Hospitalized, n (%)** | 160 (88.5%) | 1,652 (79.6%) | 0.003 |
| **Patients Prescribed Steroids, n (%)** | 71 (39.3%) | 459 (22.1%) | <0.001 |

*IBD: Inflammatory Bowel Disease, SD: Standard Deviation, n: number, CD: Crohn’s Disease, UC: Ulcerative Colitis, AP: Acute Pancreatitis, CP: Chronic Pancreatitis. P value column represents a comparison between Pediatric and Adult cohorts within each dataset.*
