# Supplementary material for: Temporal Analysis of Inflammatory Bowel Disease and Pancreatitis Co-Occurrence in Children and Adults in the United States
Source: Clin Transl Gastroenterol. 2023 Aug 9;14(11):e00628. doi: 10.14309/ctg.0000000000000628 (PMC10684167; doi:10.14309/ctg.0000000000000628)
Supplement: Supplementary file 2 [file ct9-14-e00628-s002.pptx]

## Slide 1
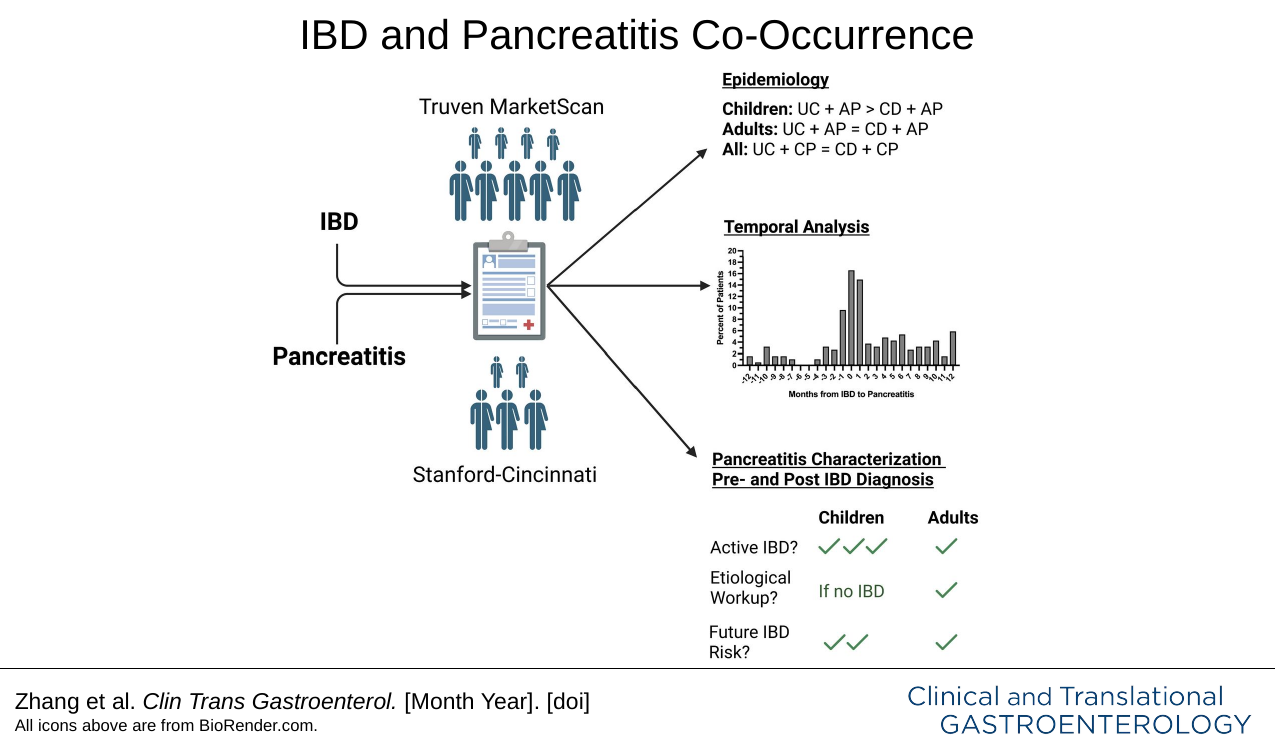

IBD and Pancreatitis Co-Occurrence
Zhang et al. Clin Trans Gastroenterol. [Month Year]. [doi]
All icons above are from BioRender.com.
